# Interrupted Time Series Analysis in Environmental Epidemiology: A Review of Traditional and Novel Modeling Approaches

**DOI:** 10.1007/s40572-025-00517-3

**Published:** 2025-12-01

**Authors:** Yiqun Ma, Tarik Benmarhnia

**Affiliations:** 1 Scripps Institution of Oceanography, University of California San Diego, 9500 Gilman Drive, La Jolla, CA 92093 USA; 2 Irset Institut de Recherche en Santé, Environnement et Travail, UMR-S 1085, University of Rennes, EHESP, Rennes, France

**Keywords:** Interrupted time-series, Quasi-experimental design, Causal inference, Environmental epidemiology, Extreme weather events

## Abstract

**Purpose of Review:**

Interrupted time series (ITS) designs are increasingly used in environmental health to evaluate impacts of extreme weather events or policies. This paper aims to introduce traditional and contemporary ITS approaches, including machine learning algorithms and Bayesian frameworks, which enhance flexibility in modeling complex temporal patterns (e.g., seasonality and nonlinear trends) and spatially heterogeneous treatment effects. We present a comparative analysis of methods such as ARIMA, machine learning models, and Bayesian ITS, using a real-world case study: estimating excess respiratory hospitalizations during the 2018 wildfire smoke event in San Francisco.

**Recent Findings:**

Our study demonstrates the practical application of these methods and provides a guide for selecting and implementing ITS designs in environmental epidemiology. To ensure reproducibility, we share annotated datasets and R scripts, allowing researchers to replicate analyses and adapt workflows. While focused on environmental applications, particularly acute exposures like wildfire smoke, the framework is broadly applicable to public health interventions.

**Summary:**

This work advances ITS methodology by integrating contemporary statistical innovations and emphasizing actionable guidance for causal inference in complex, real-world settings.

**Supplementary Information:**

The online version contains supplementary material available at 10.1007/s40572-025-00517-3.

## Introduction

Inferring causal effects of a well-defined intervention on a given health outcome is a key goal for epidemiologists, and various study designs can be used to facilitate this task. Study designs based on randomization have been recommended as a key identification strategy as they can help deal with systematic observed and unobserved differences (that can notably lead to confounding bias) between the group(s). Different types of randomized designs have been proposed, such as traditional individual or cluster parallel designs that capitalize on random treatment assignment across units at baseline [1]. In parallel, other randomized designs, such as stepped wedge designs, capitalize on the random timing of treatment assignment [2]. Indeed, in stepped wedge trials, all individuals eventually receive the intervention but at staggered, randomly determined times. Then, if the timing at which health outcomes change is correlated with the timing at which the intervention has been received, it will be possible to infer that this change is attributable to the intervention. However, in many settings, the implementation of such experimental designs is not feasible due to economic, scale, or ethical objections. This is typically the case when analyzing the health effects of environmental exposures such as environmental policies, industrial accidents, or natural hazards [3, 4]. Sometimes, the intervention of interest has already been implemented and alternative strategies to identify the target causal effect using observational data become necessary. In this context, relying on natural experiments and using quasi-experimental methods constitutes a valuable alternative approach. Such identification strategies based on observational data can be seen as emulating randomized designs or trials [5]. There are different types of natural experiments that can be capitalized on including (1) those based on eligibility for a specific intervention (treatment or exposure) and (2) those based on the timing of the intervention.

Quasi-experimental methods based on the timing of a natural experiment have been shown to be particularly useful in policy evaluation and environmental epidemiology applications [6, 7]. Such methods focus on analyzing time series of a given health outcome of interest and aim at estimating a counterfactual trend after the implementation of an intervention. Different types of methods that rely on one or multiple control (unexposed) groups have been proposed in the past few decades including canonical difference-in-differences (DID) and extensions such as synthetic control methods [8]. Yet, in many settings, control groups are not available (e.g., due to data availability) and Interrupted Time Series (ITS) methods have been proposed as a valuable approach. ITS methods can be used when multiple measurements for the outcome are available before and after a well-defined intervention and when acute changes in the outcome of interest are expected [9, 10]. The overall intuition is to approximate the counterfactual trend after the intervention (i.e., had the intervention not taken place) so such counterfactual trends can be compared to the observed trends after the intervention. In such settings, we typically aim at estimating the average treatment effect on the treated. As with other quasi-experimental methods capitalizing on the timing of natural experiments, some identification assumptions are needed. First, the parallel trend assumption (usually used for DID) requires that, in the context of ITS analyses, the modeled counterfactual trend before the intervention correctly mimics the observed trend of the outcome before the intervention. That could be done by modeling time trends (which can vary in complexity, see details below) and using time-varying covariates that predict changes in the outcome of interest and are also measured after the intervention. By design, ITS analyses adjust for any time-invariant confounding. Second, the common shock assumption requires that no other interventions that can directly or indirectly affect the outcome occurred at the same time as the intervention of interest. This assumption requires qualitative knowledge regarding the context in which the intervention took place (to ensure no other event took place around the same time) as it is untestable empirically. For ITS, as there is no control group that can help ensure that no other event may influence the outcome, this assumption is particularly important to check. Besides these two assumptions, other identification assumptions include no interference, consistency, no model misspecifications, no measurement error, and no selection bias.

A few reviews and tutorials have already introduced ITS or described how to implement them in various settings [11–16]. However, most of these existing resources have focused on traditional settings (e.g., one-stage ITS, simple temporal trends, and no consideration of potential heterogeneous effects across treated units) and did not cover most recent developments in the ITS literature. There are various approaches for ITS that have been proposed in the past decades with recent developments to consider more complex time trends, functional forms, and autocorrelation. For example, there are various options to model time trends (i.e., seasonality and long-term trends) including autoregressive integrated moving average (ARIMA) model and more recent machine learning (ML) algorithms [17, 18] and to model spatial dependency, such as the Bayesian framework [19], but comparing their results in a real case study has not been done yet. In addition, previous tutorials have not provided a comprehensive guide to help select the relevant ITS approach when inferring the causal effect of a given intervention.

In this paper, we aim at introducing several ITS approaches by a real case study where we estimate the excess respiratory hospitalizations during the 2018 wildfire smoke event in San Francisco in California. In this paper, we focus on a single intervention and do not consider settings with staggered interventions, which are discussed elsewhere [20]; although we focus on environmental applications, ITS can be applied to a large number of other topics as well [12].

## Overview of the Illustrative Case Study

### The 2018 Wildfire Smoke Event in San Francisco

**Fig. 1:**
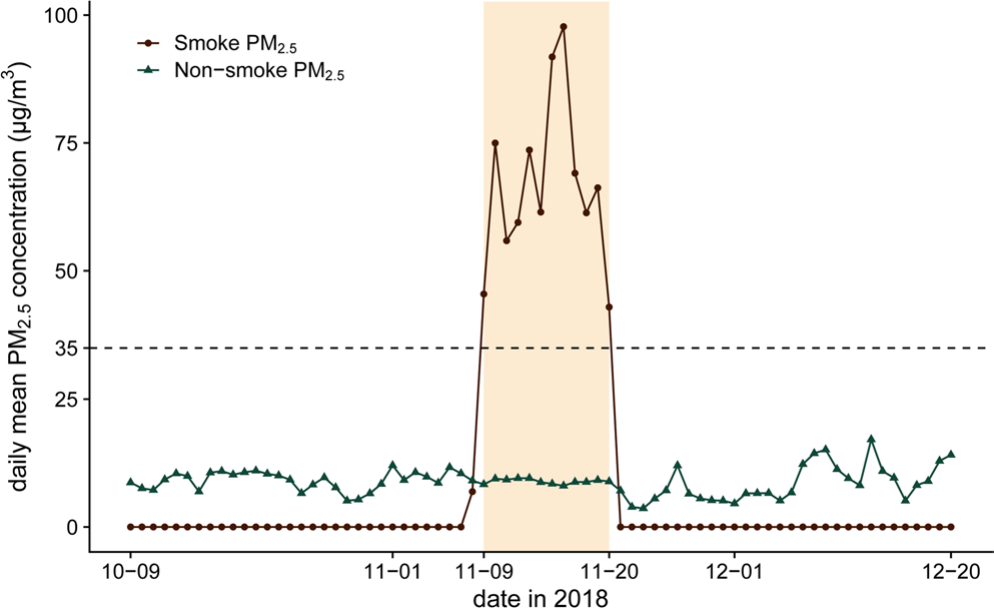
Daily mean PM_2.5_ concentrations in San Francisco, before, during, and after the 2018 wildfire smoke event. This figure shows the time series of the daily mean wildfire smoke and non-smoke PM_2.5_ concentrations (µg/m^3^) in San Francisco County, before, during, and after the 2018 wildfire smoke event. The horizontal dashed line represents the U.S. Environmental Protection Agency (EPA)’s 24-hour average standard for PM_2.5_ (35 µg/m^3^). The orange rectangle marks the period of the smoke event (November 9 to 20, 2018), when the daily mean total PM_2.5_ concentrations exceeded the EPA’s standard

On November 8, 2018, the Camp Fire ignited in Butte County, Northern California, and burned for more than two weeks [21]. The City and County of San Francisco, although located over 150 miles southwest of the ignition source and rarely threatened by wildfires, was substantially influenced by smoke from the Camp Fire. The daily mean fine particulate matter (PM_2.5_) concentrations in San Francisco exceeded 35 µg/m^3^ for 12 consecutive days from November 9 to 20, 2018, with a peak of 105 µg/m^3^ (Fig. 1). For context, 35 µg/m^3^ is the level of the U.S. Environmental Protection Agency 24-hour PM_2.5_ primary standard, defined as the 98th percentile of daily concentrations averaged over three years.

In this paper, we estimate the excess respiratory hospitalizations, defined as the difference between observed and counterfactual respiratory hospitalization counts, during this 12-day wildfire smoke event in San Francisco, and use this as a case study to illustrate the application of four different ITS models. Following the guidelines proposed by Bernal et al. (2017) [22], this case is well-suited for an ITS design because: (1) the event occurred at a clear time point, so that the pre-event and event periods can be clearly defined; (2) respiratory hospitalizations are an acute outcome that is expected to respond quickly to the onset of smoke exposure, and (3) daily hospitalization data are available for San Francisco from 2011 to 2018, providing sufficient pre- and post-event coverage. For simplicity, we assume that the smoke event had an immediate impact on respiratory hospitalizations, and that this impact diminished promptly after the event ended. However, the ITS methods presented in this review can be readily adapted to account for potential lagged effects by extending the definition of the event period.

### Overview of the ITS Models

In this review, we estimate the excess respiratory hospitalizations during the 2018 wildfire smoke event in San Francisco using four ITS modeling approaches: (1) a classical segmented regression, (2) a two-stage approach using ARIMA models, (3) a two-stage approach incorporating ML models, and (4) a Bayesian hierarchical model. We begin with the classical segmented regression model [22], the simplest form of ITS analysis. Next, we implement an ARIMA model to forecast respiratory hospitalizations in the absence of the smoke event [17], representing the counterfactual scenario. Third, we use a hybrid ML model that combines the Prophet al.gorithm with XGBoost (Prophet-XGBoost) to capture non-linear relationships and complex interactions among predictors to improve the accuracy of counterfactual prediction [18]. Although ZCTA-level data are available, we use aggregated county-level data for these three designs because only county-level datasets can be shared publicly. Finally, we apply a Bayesian hierarchical framework to account for both spatial and temporal dependencies in the data; we use ZCTA-level data for this analysis to demonstrate the spatial variations within the county [19].

We do not intend to determine which approach is the “best”; instead, by applying different ITS models to the same research question, we aim to compare and summarize the similarities and differences in their designs, implementations, and estimates.

### Analytical Dataset

The structure and sample rows of the county-level analytical dataset are shown in Table 1. Time indicators (the number of days since the start of the study, day of the year, and day of the week) and environmental variables (non-smoke PM_2.5_, mean air temperature, relative humidity, and precipitation) were used as predictors in all ITS models presented in this review. The data sources of these variables are described in Supplementary Methods 1.

**Table 1:** Structure and sample rows of the county-level analytical dataset

Date	Time	Doy	Dow	Event	Hospitalization	Non-smoke PM_2.5_	Temperature	RH	Precipitation
2011-01-01	1	1	7	0	85	9.5	9.6	76.6	4.8
2011-01-02	2	2	1	0	87	8.2	9.2	82.5	13.0
…									
2018-11-09	2870	313	6	1	63	8.3	13.3	49.8	0.0
2018-11-10	2871	314	7	1	58	9.4	12.5	57.0	0.0
…									
2018-11-20	2881	324	3	1	66	8.9	13.5	68.8	1.2
2018-11-21	2882	325	4	0	48	7.1	15.0	67.6	36.1
…									
2018-12-31	2922	365	2	0	64	7.7	10.6	80.1	0.0

This table shows the data structure of the dataset we used for the case study.

Time: the time index from the first to the last day of the dataset.

Doy: day of the year.

Dow: day of the week (Sunday to Saturday, 1–7).

Event: the indicator of the smoke event from 2018-11-09 to 2018-11-20 (0 = no, 1 = yes).

Hospitalization: daily respiratory hospitalization counts in San Francisco.

Non-smoke PM_2.5_: daily non-smoke PM_2.5_ concentration (µg/m^3^).

Temperature: daily mean air temperature (˚C).

RH: daily mean relative humidity (%).

Precipitation: daily total precipitation (mm).

This study was approved by the California Health and Human Services Agency’s Committee for the Protection of Human Subjects (project number: 2021 − 116). All statistical analyses were conducted with R software (version 4.3.1). The county-level datasets and R scripts used in this study are publicly available at https://github.com/benmarhnia-lab/ITS_review.

## Classical ITS Design with Segmented Regression

In the classical ITS analysis, we implemented the following segmented regression using a quasi-Poisson model:

1 $$\begin{array}{l}\log\left(E\left[Y_t\right]\right)=\beta_0+\beta_1Event_t+\beta_2Time_t\\+\sum_{k=1}^2\left(a_k\cos\left(\frac{2\pi kDoy_t}{365}\right)+Y_k\sin\left(\frac{2\pi kDoy_t}{365}\right)\right)\\+\delta Dow_t+\beta_3NonsmokePm2.5_t+ns\left(Tmean_{t,}\;df=3\right)\\+\beta_4RH_t+\beta_5Precipitation_t\end{array}$$

where $\:{Y}_{t}$ is the daily count of respiratory hospitalizations on day $\:t$, and $\:{Event}_{t}$ is a binary indicator for the smoke event (coded 1 during the event period and 0 otherwise). $\:{Time}_{t}$ represents the number of days since the start of the study. Seasonality was modeled using harmonic terms of day of the year ($\:{Doy}_{t}$). Day-of-week effects ($\:{Dow}_{t}$, a categorical variable) were included to account for weekly patterns in hospitalizations. We also adjusted for daily non-smoke PM_2.5_ concentrations ($\:{NonsmokePM2.5}_{t}$), mean air temperature ($\:{Tmean}_{t}$, modeled using a natural cubic spline with 3 degrees of freedom [dfs]), mean relative humidity ($\:{RH}_{t}$), and total precipitation ($\:{Precipitation}_{t}$) in the model.

Here, $\:{\beta\:}_{0}$ is the intercept; $\:{\beta\:}_{1}$ estimates the immediate level change associated with the smoke event; $\:{\beta\:}_{2}$ captures the long-term time trend in hospitalizations; and $\:{\beta\:}_{3}$, $\:{\beta\:}_{4}$, and $\:{\beta\:}_{5}$ are the coefficients for the associations with non-smoke PM_2.5_, relative humidity, and precipitation. We did not include an interaction between $\:{Event}_{t}$ and $\:Time$ in the main model, assuming no change in slope over the short event period.

In secondary analyses, we considered alternative numbers of dfs (4 and 5) for air temperature, added an interaction between $\:{Event}_{t}$ and $\:{Time}_{t}$, used month-of-year instead of day-of-year in the harmonic terms, and used a natural cubic spline with 3 dfs for $\:{Time}_{t}$. The main model had the lowest quasi-Akaike Information Criterion (QAIC) value among all specifications (Supplementary Table 1).

**Fig. 2:**
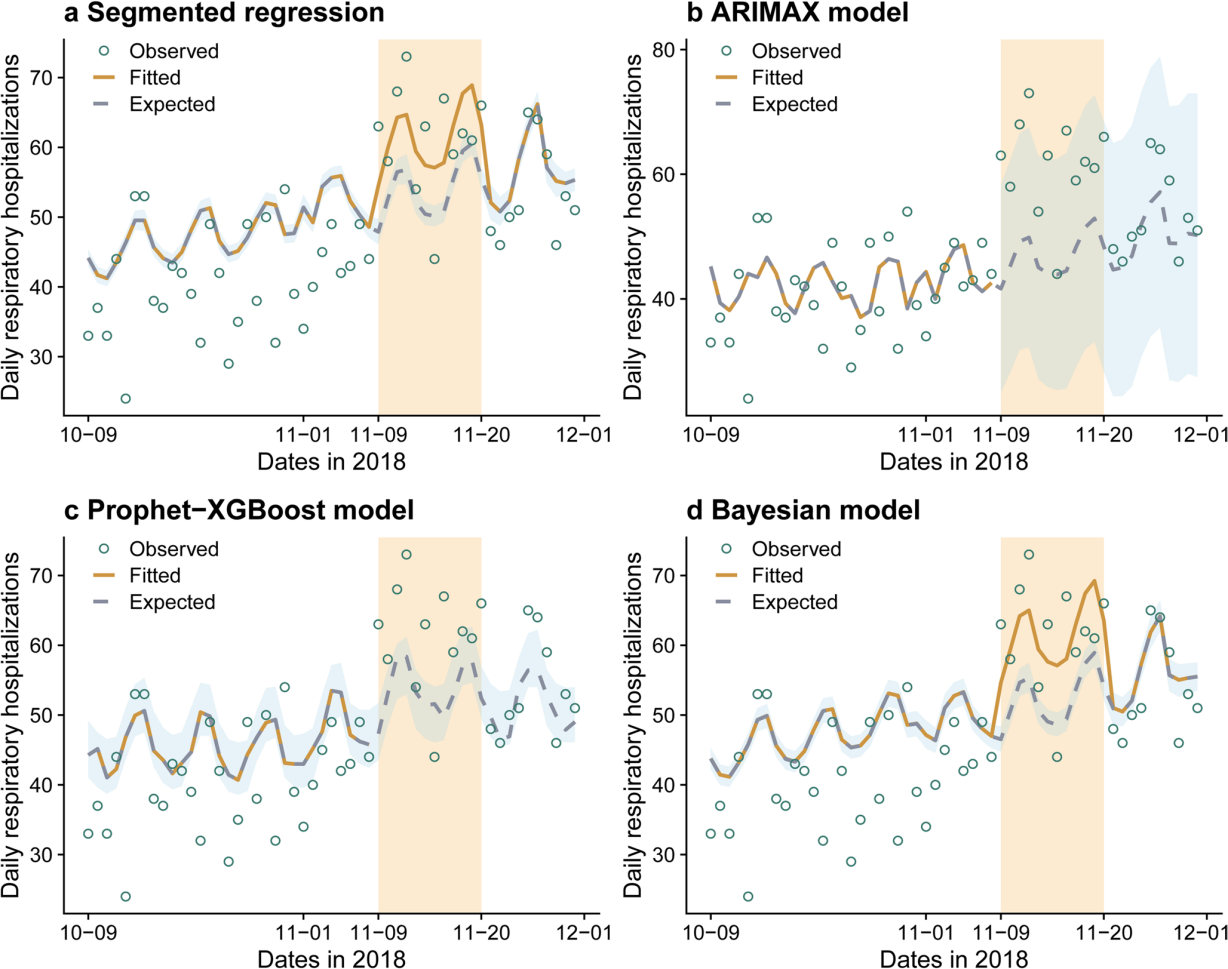
Daily observed, fitted, and expected respiratory hospitalizations from different ITS modeling approaches. This figure displays the daily observed counts of respiratory hospitalizations (green circles), the fitted values (yellow curve) and the expected values under the counterfactual scenario in which the event did not occur (dashed grey curve), from the segmented regression (**a**), ARIMAX model (**b**), Prophet-XGBoost model (**c**), and Bayesian ITS model (**d**). The event period is highlighted by the orange rectangle. The fitted and expected curves overlap before (a-d) and after (b and c) the event period. The light blue shaded areas represent the 95% CIs or PIs of the expected values

Figure 2a presents the results from the classical ITS analysis using segmented regression, in which the dashed grey curve indicates the trend under counterfactual scenario in which the smoke event did not occur (i.e., with the event indicator set to 0 during the event period). This model estimated a 14% increase in respiratory hospitalizations during the smoke event period (relative risk [RR]: 1.14; 95% confidence interval [CI]: 1.03, 1.26), corresponding to an estimated 90 (95% CI: 69, 110) excess hospitalizations.

## Two-stage ITS Analysis Using ARIMA Models

Segmented regression may not be suitable in all cases, particularly when it is challenging to adequately model the autocorrelation structure of the outcome (e.g., detected by Durbin-Watson test, Breusch-Godfrey test, Ljung-Box Q-test, or correlograms). As an alternative, ARIMA models can be used to account for temporal dependence in time-series data [17]. Different from segmented regressions, ARIMA models explicitly incorporate autoregressive and moving average components, allowing more accurate modeling and forecasting of outcomes that exhibit serial correlation.

In this case study, we applied an Autoregressive Integrated Moving Average with Exogenous variables (ARIMAX) model, an extension of the ARIMA model that incorporates external regressors. External regressors are consistent with those used in the segmented regression described in Eq. (1). We demonstrate a two-stage approach. In the first stage, we fitted an optimized model to pre-event data; in the second stage, we applied this model to post-event predictors to forecast expected respiratory hospitalizations during and after the event period under the counterfactual scenario of no smoke event.

We used the automated model selection algorithm in the *forecast* R package to identify the optimal parameters ($\:p,d,q$) of the ARIMAX model by minimizing the corrected Akaike information criterion (AICc). $\:p$ denotes the order of the autoregressive (AR) component, $\:d$ is the degree of non-seasonal differencing, and $\:q$ is the order of the moving average (MA) part of the model. The selected model was ARIMAX(2, 1, 3), which can be expressed as follows:

2 $$\:\begin{array}{c}{\triangle Y}_t=\:Y_t-\:Y_{t-1}\end{array}$$

3 $$\begin{array}{l}{\triangle Y}_t=\:X_t^{\top\:}\beta\:+\:{\varphi\:}_1{\triangle Y}_{t-1}+\:{\varphi\:}_2{\triangle Y}_{t-2}\\+\epsilon_t+{\theta\:}_1\epsilon_{t-1}+{\theta\:}_2\epsilon_{t-2}+{\theta\:}_3\epsilon_{t-3}\end{array}$$

where $\:{\varDelta\:Y}_{t}$ is the first-differenced outcome to ensure stationarity, $\:{X}_{t}^{\top\:}\beta\:$ is the linear combination of all covariates, $\:{\varphi\:}_{1}$ and $\:{\varphi\:}_{2}$ are autoregressive coefficients, and $\:{\theta\:}_{1}$, $\:{\theta\:}_{2}$, and $\:{\theta\:}_{3}$ are moving average coefficients. $\varepsilon_t$ is the error term. In other words, $\:{\varDelta\:Y}_{t}$ is modeled as a function of 2 lagged values of $\:{\varDelta\:Y}_{t}$, 3 lagged values of $\varepsilon_t$, and external covariates. The automated algorithm did not recommend a seasonal model, likely due to the use of first-order differencing and the inclusion of harmonic terms that captured seasonal variation. The residual diagnostic plots suggest that the model fits the pre-event data well, with no apparent patterns in the residuals, no significant remaining autocorrelation, and an approximately normal distribution of the residuals (Supplementary Fig. 1).

Then, we applied this model to forecast respiratory hospitalizations during and after the event. The uncertainty was summarized with 95% prediction intervals (PIs), the range within which a future value would fall with 95% probability, conditional on the selected model. PIs can be obtained analytically by assuming the forecast errors follow a Gaussian distribution with a variance estimated from the model residuals, or via bootstrapping by resampling the historical residuals of the model to simulate future sample paths and taking empirical quantiles. In this study, we report the parametric PIs in the main text and provide the bootstrapped intervals (1,000 times) in Supplementary Fig. 2. The interval widths were similar under both methods in our example.

Because the daily county-level respiratory hospitalization counts in this case study are relatively large and not bounded by zero, they are suitable for modeling using traditional ARIMA models, which assume approximately normally distributed residuals [17]. Therefore, we directly applied the classical ARIMA framework in the main analysis to demonstrate the use of automated model selection. However, extensions of ARIMA to generalized linear models have been developed to better accommodate count data and distributional assumptions [23, 24]. As a secondary analysis, we fitted the same ARIMAX model structure using a Poisson regression implemented via the *tsglm* package in R (Supplementary Table 2) [23]. While both models produce similar results in our example, this consistency may not hold in other cases, in which the Poisson regression may provide better variance estimation.

The in-sample error measures for the ARIMAX forecasting model are summarized in Supplementary Table 3. Figure 2b displays the forecasted respiratory hospitalization counts from the model. We estimated approximately 174 excess hospitalizations during the smoke event, with wide uncertainty (95% PI: −47 to 394).

## Two-stage ITS Analysis with ML Models

Both segmented regression and ARIMA models assume that time trends in the pre- and post-event periods can be expressed as a linear combination of parameters [25]. Although researchers can include interaction and non-linear terms in the model, if the true relationship between the outcome and predictors is more complex than what the model can capture, ITS analyses may produce biased estimates [25]. Motivated by this limitation, researchers have started integrating ML approaches into the ITS framework [26, 27]. Many ML algorithms can accommodate non-linear interactions among covariates and complex functional forms. They have demonstrated strong performance in modeling trends through data-driven processes, often outperforming linear models in predictive accuracy [28].

In this case study, we applied Prophet-XGBoost as the predictive model. Prophet is an additive regression model for forecasting time series data [29]. XGBoost is a powerful gradient boosting framework that models complex, non-linear relationships and interactions among covariates [30]. In the hybrid model, Prophet decomposes the time series into long-term trend, seasonality, and holiday effects, and XGBoost models the residuals from the Prophet model through an ensemble of decision trees. We used the Prophet-XGBoost model as an example in this study, but other ML algorithms can also be applied in the two-stage ITS design.

Predictors included time-related features (days since the start of the study, year, month, quarter, day-of-the-week, day-of-the-month, day-of-the-year, harmonic terms of day of the year, etc.) and environmental variables up to 7 lag days (non-smoke PM_2.5_, mean air temperature, relative humidity, and precipitation). Unlike traditional regression models, there was no need to pre-specify non-linear transformations for variables such as temperature, as the ML algorithm can automatically capture non-linear effects. All relevant time features and lagged environmental predictors were included with limited concern for multicollinearity, since the tree-based XGBoost component of the model is relatively robust to correlated inputs and can selectively partition the feature space based on predictive relevance when the sample size is large.

We divided the dataset into three subsets: a training period (January 1, 2011, to June 30, 2018), a testing period (July 1, 2018, to November 7, 2018), and a post-event period (during and after the smoke event; November 9, 2018, to December 31, 2018). We applied time-series cross-validation on the training data using a sliding window approach [31, 32]. A total of 5 folds with a non-overlapping validation window were created. Each fold contained a training period of 2 years followed by a 12-month testing period (Supplementary Fig. 3). Both the Prophet and XGBoost components were tuned jointly over a space-filling grid of 100 hyperparameter combinations. The Prophet parameters tuned included growth type, changepoint range, and prior scales for seasonality and changepoints. The XGBoost parameters tuned included tree depth, learning rate, number of predictors sampled at each split, minimum samples per node, loss reduction, and early stopping iterations. The optimal parameter set is determined by minimizing the Root Mean Square Error (RMSE) across the cross-validation folds. To quantify prediction uncertainty, we employed a Moving Block Bootstrap approach with a block size of 14 days (*n* = 1,000). The 95% empirical CIs (eCIs) were derived from the empirical distribution of these 1,000 predictions, taking the 2.5^th^ and 97.5^th^ percentiles as the lower and upper bounds, respectively. Statistical analyses were conducted with the *tidymodels* and *modeltime* R packages.

Overall, the model performed well in both training and testing sets, with an RMSE of 6.84 and 6.50, respectively (Supplementary Table 4). The model predicted a total of 643 (95% eCI: 594, 690) expected respiratory hospitalizations during the smoke event period, under the counterfactual scenario, resulting in a total of 95 (95% eCI: 48, 144) excess respiratory hospitalizations during the smoke event (Fig. 2c).

## ITS within a Bayesian Framework

In addition to the complex temporal trends, another important consideration in ITS analysis is the spatial patterns in data. Capturing spatial variations in the health impacts of exposure events is essential for understanding disparities in population health, guiding resource allocation, and designing targeted public health interventions. With panel data spanning multiple spatial units, all the aforementioned approaches can be used to estimate event impacts for each unit (e.g., by adding random intercepts for spatial units or running models for each unit separately); however, unless explicitly modeled, they may fail to account for spatial dependency. To account for both temporal and spatial dependencies in data, researchers proposed ITS models within a Bayesian hierarchical framework [19].

In this case study, using ZCTA-level data, we implemented the following model with a Poisson regression:

4 $$\begin{array}{l}\log\left(E\left[Y_{i,\;t}\right]\right)=\beta_0+\beta_1Event_t+\beta_2Time_t\\+\sum_{k=1}^2\left(a_k\cos\left(\frac{2\pi kDoy_t}{365}\right)+Y_k\sin\left(\frac{2\pi kDoy_t}{365}\right)\right)\\+\delta Dow_t+\beta_3NonsmokePM2.5_{i,\;t}\\+ns\left(Tmean_{i,\;t},\;df=3\right)+\beta_4RH_{i,\;t}\\+\beta_5Precipitation_{i,\;t}+\:{u}_{month\left(t\right)}+v_i+\in_{i,\;t}\end{array}$$

where $\:{Y}_{i,t}$ is the daily count of respiratory hospitalizations in ZCTA $\:i$ on day $\:t$. $\:{u}_{month\left(t\right)}$ is the temporal random effect over month and $\:{v}_{i}$ is the spatial random effect over ZCTAs. Note that here we use these two random effects as an example, but researchers can decide the spatial and temporal units for the random effects based on their specific cases. All other covariates are consistent with those listed in Eq. (1). 

We followed the prior specification described in Gascoigne (2024) [19]. In brief, we specified weakly informative normal priors for the intercept and fixed-effect parameters, with a mean of 0 and variance of 1,000. The temporal random effect was modeled as a first-order random walk (RW1). The spatial random effect was modeled using the Besag-York-Mollié 2 (BYM2) model, which represents a weighted combination of a structured component that captures spatial dependence among neighboring areas and an unstructured component that accounts for independent heterogeneity [33]. We used penalized complexity (PC) priors to regularize the variance components of the random effects [34]. For both temporal and spatial random effects, we set a PC prior such that the probability of the standard deviation exceeding 1 was 0.01. Additionally, for the spatial random effect, the mixing parameter ($\:\varphi\:$) has a PC prior where $\:\text{Pr}\left(\varphi\:>\frac{1}{2}\right)=\frac{2}{3}$, which moderately favor unstructured over structured spatial variation.

To estimate expected hospitalizations under the counterfactual scenario, we generated posterior samples of the model parameters (*n* = 1,000) and predicted outcomes setting the event indicator to zero while keeping all other covariates unchanged. The posterior mean and 95% credible intervals of predicted values were computed across all samples. This ITS model within a Bayesian hierarchical framework was fitted using Integrated Nested Laplace Approximation (INLA), the *INLA* package in R [35].

**Fig. 3:**
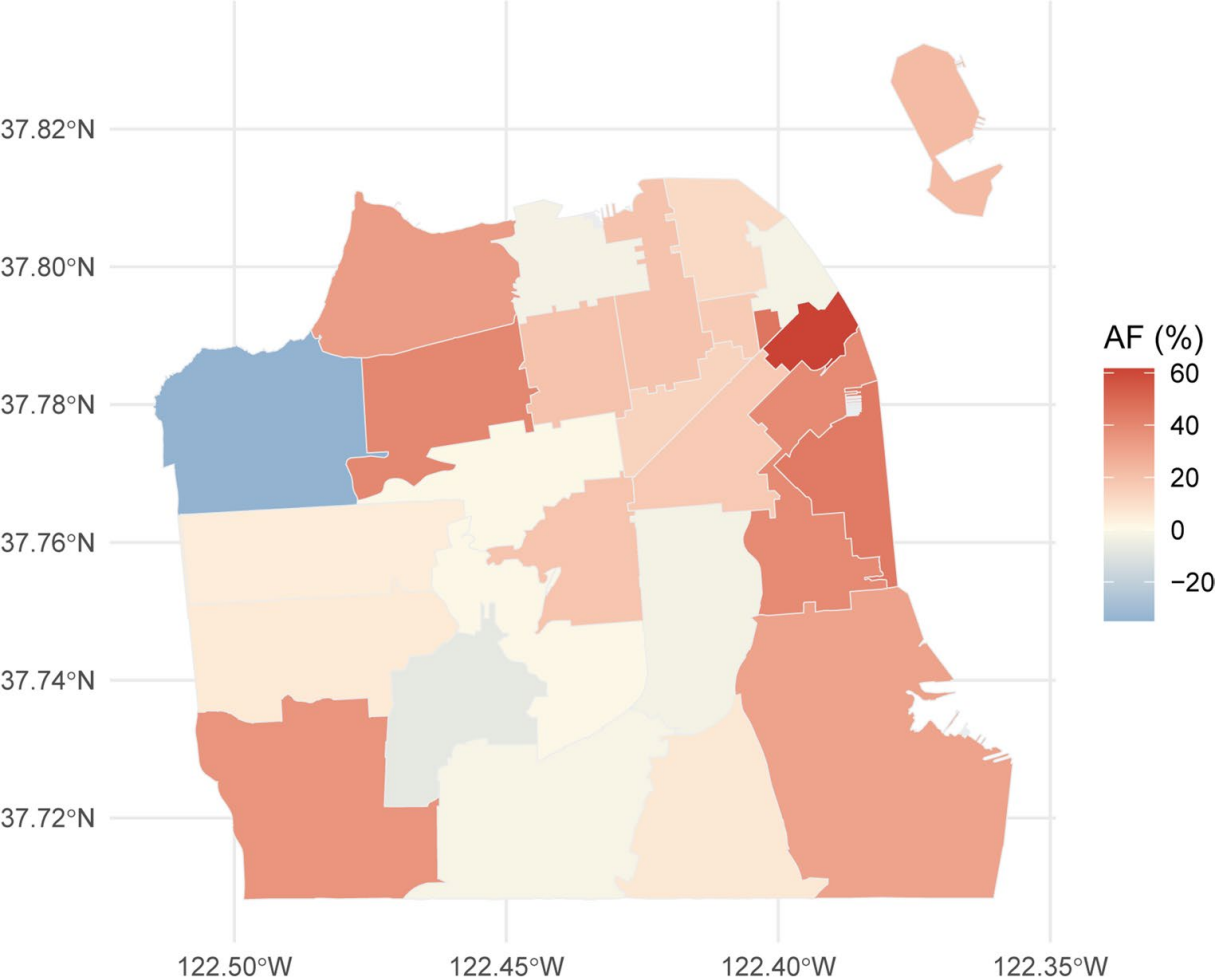
ZCTA-level respiratory hospitalizations attributable to the smoke event estimated by the Bayesian ITS model. This map displays the attributable fraction (AF) of respiratory hospitalizations to the smoke event in each ZCTA in San Francisco

The fitted and estimated expected hospitalizations are shown in Fig. 2d. This model estimates a total of 109 (95% eCI: 87, 131) excess respiratory hospitalizations during the smoke event, with an RR of 1.17 (95% CI: 1.09, 1.26) for the event. Figure 3 displays the attributable fraction (AF; AF = [excess hospitalizations/observed hospitalizations] × 100%) of respiratory hospitalizations to the smoke event in each ZCTA in San Francisco. Higher AFs were observed along the eastern waterfront and southern Bayview region, whereas the AFs were close to 0 or slightly negative in central ZCTAs and western neighborhoods (e.g., Richmond district). These ZCTA-specific estimates provide a foundation for probing the drivers of the spatial heterogeneity, such as topography, the built environment, and socioeconomic conditions.

## Discussion

In this review, we introduced different approaches for conducting ITS analyses and compared the results using excess respiratory hospitalizations during the 2018 wildfire smoke event in San Francisco as a case study. The segmented regression (90; 95% CI: 69, 110), Prophet-XGBoost (95; 95% eCI: 48, 144), and the Bayesian hierarchical model (109; 95% CI: 87, 131) all estimated about 100 excess hospitalizations; the central estimate from the ARIMAX model was higher (174), but with wide 95% PIs (−47, 394). Based on the Cochran’s Q test, there is no evidence of heterogeneity among these four estimates (*P* = 0.57) (Supplementary Table 5).

We provide a detailed methodological comparison in Table 2. Both the classical segmented regression and Bayesian ITS frameworks require an explicit event indicator, specified based on a pre-assumed model of the event’s impact. The counterfactual is then generated by setting this indicator to 0 while holding all other covariates constant. In contrast, two-stage approaches, such as two-stage ARIMA ITS and two-stage ML ITS, do not rely on a predefined impact model. Instead, they estimate the counterfactual using purely predictive models trained on pre-event data. Compared to one-stage models, two-stage ITS designs naturally allow for time-varying effects of the event, inferred from the daily difference between observed and predicted outcomes. Although one-stage approaches can also yield daily excess estimates, the estimated event effect is typically constant over time unless explicitly modeled using interactions or time-varying structures. It is important to note that ARIMA and ML models can also be implemented in a one-stage ITS framework by including an event indicator and fitting the model to the full dataset (both pre- and post-event); likewise, generalized linear models and Bayesian hierarchical models can be adapted to a two-stage ITS design by restricting model fitting to pre-event data and generating post-event counterfactual predictions accordingly.

**Table 2:** Comparison of different ITS models

	Classical ITS	Two-stage ARIMA ITS	Two-stage ML ITS	Bayesian ITS
How to generate the counterfactual	Make predictions by setting event = 0 while holding all other covariates constant	Fit the model using pre-event data and forecast the outcome using the ARIMA structure and external regressors that are unrelated to the event	Train the model using pre-event data and predict the outcome using predictors that are unrelated to the event	Make predictions by setting event = 0 while holding all other covariates constant
How to handle seasonality and complex temporal trend	Manual specification	Differencing and seasonal ARIMA component	Automatic adaptation to complex, nonlinear, and evolving trends	Manual specification and temporal random effects
How to account for time-varying covariates	Directly include in the model	Include external regressors (ARIMAX)	Directly include in the predictors; learn nonlinear and interaction effects automatically	Directly include in the model
How to account for spatial autocorrelation	Not modeled explicitly	Not modeled explicitly	Not modeled explicitly	Spatial random effect
How to account for temporal autocorrelation	Not modeled explicitly; manual adjustment for seasonality or lags	ARIMA structure	Not modeled explicitly; lags and gradient boosting, time-series bootstrap	Not modeled explicitly; temporal random effect
How to validate the model (validation strength)	Visual inspection of fitted vs. observed values	Forecast accuracy metrics	Full cross-validation and out-of-sample error metrics	Posterior predictive checks
How to handle time-varying effect of event	Pre-assumed and manually specified impact model	Captured by the difference between observed and predicted values	Captured by the difference between observed and predicted values	Pre-assumed and manually specified impact model
How to measure the uncertainty	Parametric standard error (SE) from the regression	Parametric or bootstrapped prediction intervals	Bootstrapping	Bayesian posterior distributions

Seasonality and time-varying covariates can be incorporated into all ITS models through manual specification. However, classical segmented regression relies entirely on prespecified terms, while the temporal random effect in Bayesian hierarchical models can also capture the residual variation in time. ARIMA-based ITS models handle seasonality and trends through differencing and seasonal ARIMA components. ML models, such as Prophet-XGBoost, offer the greatest flexibility in modeling temporal complexity, which automatically adapt to complex, nonlinear, and evolving trends and learn nonlinear and interaction effects automatically.

Autocorrelation violates the usual independence-of-errors assumption; if left unaddressed it typically yields incorrect (typically anticonservative) standard errors [36]. Among the four ITS modeling approaches discussed in our review, only the ARIMA models explicitly incorporate temporal autocorrelation through autoregressive and moving average components [17]. Beyond ARIMA, other commonly used ways to handle autocorrelation in ITS include the Prais-Winsten procedure, a form of generalized least squares method which applies a linear transformation to the outcome and explanatory variables to decorrelate the error term [37], and Newey-West adjustment, a method for calculating heteroskedasticity- and autocorrelation-consistent standard errors in a regression model [38]. These methods have been introduced in detail and applied in the ITS context with segmented regressions in prior studies [15, 16]. In ML models (e.g., Prophet-XGBoost), serial autocorrelation can be accommodated by using time-series bootstrapping for SE estimation (e.g., moving, circular, or stationary block bootstrap), and indirectly by including lagged features and allowing the learning algorithm to capture residual temporal structure. Bayesian ITS can account for temporal autocorrelation by including appropriately specified temporal random effects, with posterior intervals reflecting that dependence conditional on the chosen structure. In terms of spatial autocorrelation, only the Bayesian ITS framework directly incorporates it, typically using a spatial random effect (e.g., by BYM2 model) [33]. This allows the model to borrow information from neighboring spatial units and adjust for unmeasured spatial confounding. The other three approaches do not account for spatial correlation unless explicitly modified.

In terms of model validation, the two-stage ML approach provides the strongest validation framework, with full cross-validation with expanding or rolling windows [31, 32] and quantitative error metrics on both training and testing sets, making it ideal for predictive evaluation. ARIMA models and Bayesian hierarchical models support moderate validation through forecast accuracy metrics or posterior predictive checks, though out-of-sample performance is not typically assessed. Model validation is most limited in classical segmented regression, which typically involves visual inspection of fitted vs. observed values and goodness-of-fit statistics. Finally, regarding uncertainty estimation, segmented regressions estimate uncertainty using parametric standard errors. ARIMA models can estimate uncertainty with either parametric prediction intervals or bootstrapping. In Bayesian hierarchical models, uncertainty can be measured according to the Bayesian posterior distributions. For ML models, bootstrapping can be used to estimate uncertainty, which provides empirical confidence intervals around predicted outcomes.

Each method has specific tradeoffs. Classical segmented regression models offer simplicity and great interpretability but strongly rely on correct impact model and functional forms. ARIMA models are straightforward and inherently account for autocorrelation and seasonality, but they are less flexible in modeling nonlinear or nonstationary patterns, and their predictive uncertainty can be large. ML-based approaches provide high flexibility and strong predictive performance, particularly in the presence of complex, nonlinear relationships and lagged covariate effects, but they are computational demanding, and the complexity can make it difficult for researchers to explicitly state and test the underlying assumptions [18]. Bayesian ITS framework accounts for both spatial and temporal dependencies, but at the cost of higher complexity and demands of computational resources. The choice of method should be guided by the specific research goal and priority, the size of data, and the availability of prior knowledge.

There are different settings we did not cover in this paper that we would like to acknowledge. First, we focused on a single intervention and did not cover situations with staggered or repeated interventions. For example, it is possible that a given community can be exposed to multiple wildfire smoke events or to compounded events (e.g., a heat wave can occur before, during, or after the wildfire smoke event). Recent advances in literature have proposed solutions to deal with such situations [39]. Second, we did not consider heterogenous effects of the intervention across subgroups in the affected population. In the literature of time-series analysis, simple approaches based on stratification (coupled with heterogeneity tests) [40] or by modeling an outcome that directly compares the outcomes across two or multiple groups [41] have been used and can be easily adapted to ITS settings. Third, we did not consider possible indirect effects regarding how the exposure of interest may affect the health outcome. For example, if the exposure of interest is a heatwave, it is possible to decompose the total effect into direct (temperature effects) and indirect (e.g., through an increase in tropospheric ozone) effects which has been done in traditional time-series settings [42] and can be adapted to ITS analyses. That can be also helpful for environmental policies that include multiple components and affect multiple areas to understand which components contribute the most to potential health benefits [43].

The use of ITS analyses can be particularly useful for environmental epidemiologists to analyze the acute health effects of extreme weather events or environmental policies. In this paper, we provide an overview and comparison of multiple ITS methods. We describe a step-by-step approach to guide researchers who may be interested in implementing such methods, detail features of each ITS approach, and provide datasets and R scripts to facilitate such implementation.

## Supplementary Information

Below is the link to the electronic supplementary material.

ESM 1 (PDF 628 KB)

## Data Availability

The county-level dataset used in this study is publicly available at https://github.com/benmarhnia-lab/ITS_review.

## Code Availability

The R scripts used in this study are publicly available at https://github.com/benmarhnia-lab/ITS_review.
