# Supplementary material for: Interrupted Time Series Analysis in Environmental Epidemiology: A Review of Traditional and Novel Modeling Approaches
Source: Curr Environ Health Rep. 2025 Dec 1;12(1):50. doi: 10.1007/s40572-025-00517-3 (PMC12669373; doi:10.1007/s40572-025-00517-3)
Supplement: Supplementary file 1 — (PDF 628 KB) [file 40572_2025_517_MOESM1_ESM.pdf]

Supplementary Information

Interrupted Time Series Analysis in Environmental Epidemiology: A Review of Traditional and Novel Modeling Approaches

Yiqun Ma[1], Tarik Benmarhnia[1,2]

[1] Scripps Institution of Oceanography, University of California, San Diego, CA, USA

[2] Irset Institut de Recherche en Santé, Environnement et Travail, UMR-S 1085, Inserm, University of Rennes, EHESP, Rennes, France

Table of contents

Supplementary Methods 1. Sources of hospitalization and environmental data

Supplementary Table 1. QAIC values of segmented regression with alternative model specifications

Supplementary Table 2. Excess hospitalizations estimated by the classical ARIMAX and an ARIMAX with Poisson regression

Supplementary Table 3. In-sample error measures for the ARIMAX forecasting model

Supplementary Table 4. Error metrics for the Prophet-XGBoost model in training and testing sets

Supplementary Table 5. Cochran's Q heterogeneity test for estimates from the four models

Supplementary Fig. 1. Residual diagnostic plot for the ARIMAX model

Supplementary Fig. 2. Daily observed, fitted, and expected respiratory hospitalizations from the ARIMAX model with bootstrapped prediction intervals

Supplementary Fig. 3. Cross-validation plan for the machine learning model

Supplementary Methods 1. Sources of hospitalization and environmental data

Respiratory hospitalization data from 2011 to 2018 was obtained from California Department of Health Care Access and Information [1]. We calculated daily ZIP Code Tabulation Area (ZCTA)-level and county-level respiratory hospitalization counts based on patients' residential addresses and the primary International Classification of Diseases (ICD)-9 and ICD-10 codes. The specific diagnosis codes used to identify respiratory hospitalizations are listed below:

- **ICD-9 codes**: "460", "461", "462", "463", "464", "465", "466", "470", "471", "472", "473", "474", "475", "476", "477", "478", "480", "481", "482", "483", "484", "485", "486", "487", "490", "491", "492", "493", "494", "495", "496", "500", "501", "502", "503", "504", "505", "506", "507", "508", "510", "511", "512", "513", "514", "515", "516", "517", "518", "519".
- **ICD-10 codes**: "J00", "J01", "J02", "J03", "J04", "J05", "J06", "J09", "J10", "J11", "J12", "J13", "J14", "J15", "J16", "J17", "J18", "J20", "J21", "J22", "J30", "J31", "J32", "J33", "J34", "J35", "J36", "J37", "J38", "J39", "J40", "J41", "J42", "J43", "J44", "J45", "J46", "J47", "J60", "J61", "J62", "J63", "J64" ,"J65", "J66", "J67", "J68", "J69", "J70", "J80", "J81", "J82", "J83", "J84", "J85", "J86", "J90", "J91", "J92", "J93", "J94", "J95", "J96", "J97", "J98", "J99".

Daily mean temperature, relative humidity, and total precipitation data from 2011 to 2018 at 4×4 km^2 grid were obtained from the Gridded Surface Meteorological (gridMET) reanalysis product [2]. ZCTA-level meteorological variables were extracted at the population-weighted centroids of each ZCTA in San Francisco. Daily ZCTA-level total and wildfire-specific $PM_{2.5}$ concentrations were estimated using an ensemble-based machine learning model in a previous study [3]. Non-smoke $PM_{2.5}$ concentration was calculated as the difference between total $PM_{2.5}$ and wildfire-specific $PM_{2.5}$. We aggregated the ZCTA-level environmental dataset to county level by calculating the average of the values across all ZCTAs in San Francisco.

Supplementary Table 1. QAIC values of segmented regression with alternative model specifications

Main model	QAIC
Using 4 dfs for temperature	2927.0
Using 5 dfs for temperature	2927.9
Including an interaction between event and time	2928.9
Using month-of-year in harmonic terms	2928.0
Using a natural cubic spline with 3 dfs for time	2930.0

QAIC: quasi-Akaike Information Criterion
dfs: degrees of freedom

Supplementary Table 2. Excess hospitalizations estimated by the classical ARIMAX and an ARIMAX with Poisson regression

Model	Excess hospitalizations
Main model (classical ARIMAX)	174 (-47, 394)
Poisson regression	128 (-48, 300)

Supplementary Table 3. In-sample error measures for the ARIMAX forecasting model

ME	RMSE	MAE	MPE	MAPE	MASE
-0.02	8.46	6.57	-2.43	13.18	0.56

ME: mean error
RMSE: root mean square error
MAE: mean absolute error
MPE: mean percentage error
MAPE: mean absolute percentage error
MASE: mean absolute scaled error

Supplementary Table 4. Error metrics for the Prophet-XGBoost model in training and testing sets

Set	RMSE	MAE	MAPE	SMAPE
Training	6.84	5.33	0.11	0.10
Testing	6.50	5.05	0.14	0.13

RMSE: root mean square error
MAE: mean absolute error
MAPE: mean absolute percentage error
SMAPE: symmetric mean absolute percentage error

Supplementary Table 5. Cochran's Q heterogeneity test for estimates from the four models

Model	Excess hospitalization (95% CI)	Cochran's Q	*P* value	I^2
Segmented regression	90 (69, 110)	2.00 (df=3)	0.57	0%
ARIMAX model	174 (-47, 394)			
Prophet-XGBoost model	95 (48, 144)			
Bayesian hierarchical model	109 (87, 131)			

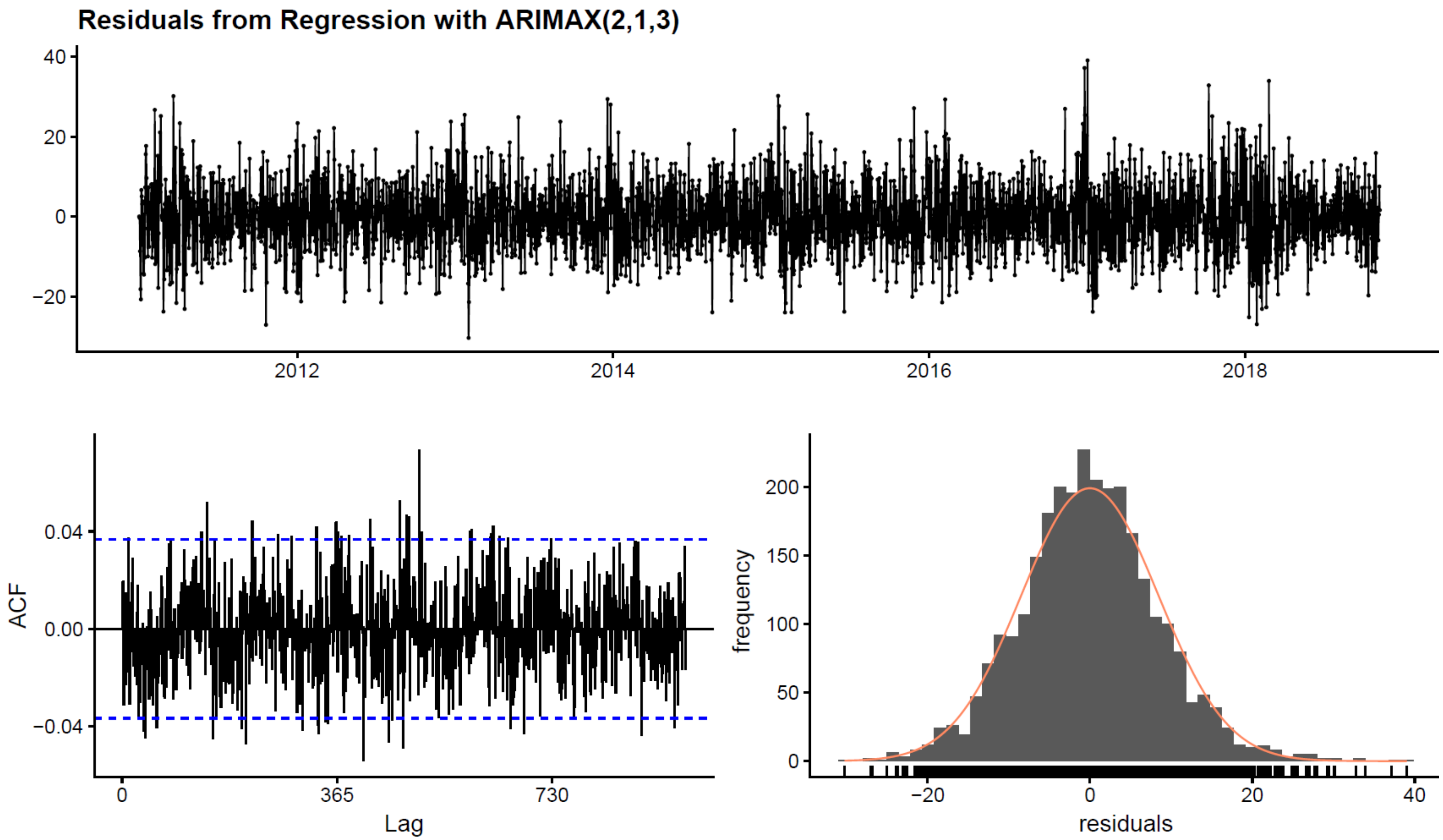

Supplementary Fig. 1. Residual diagnostic plot for the ARIMAX model

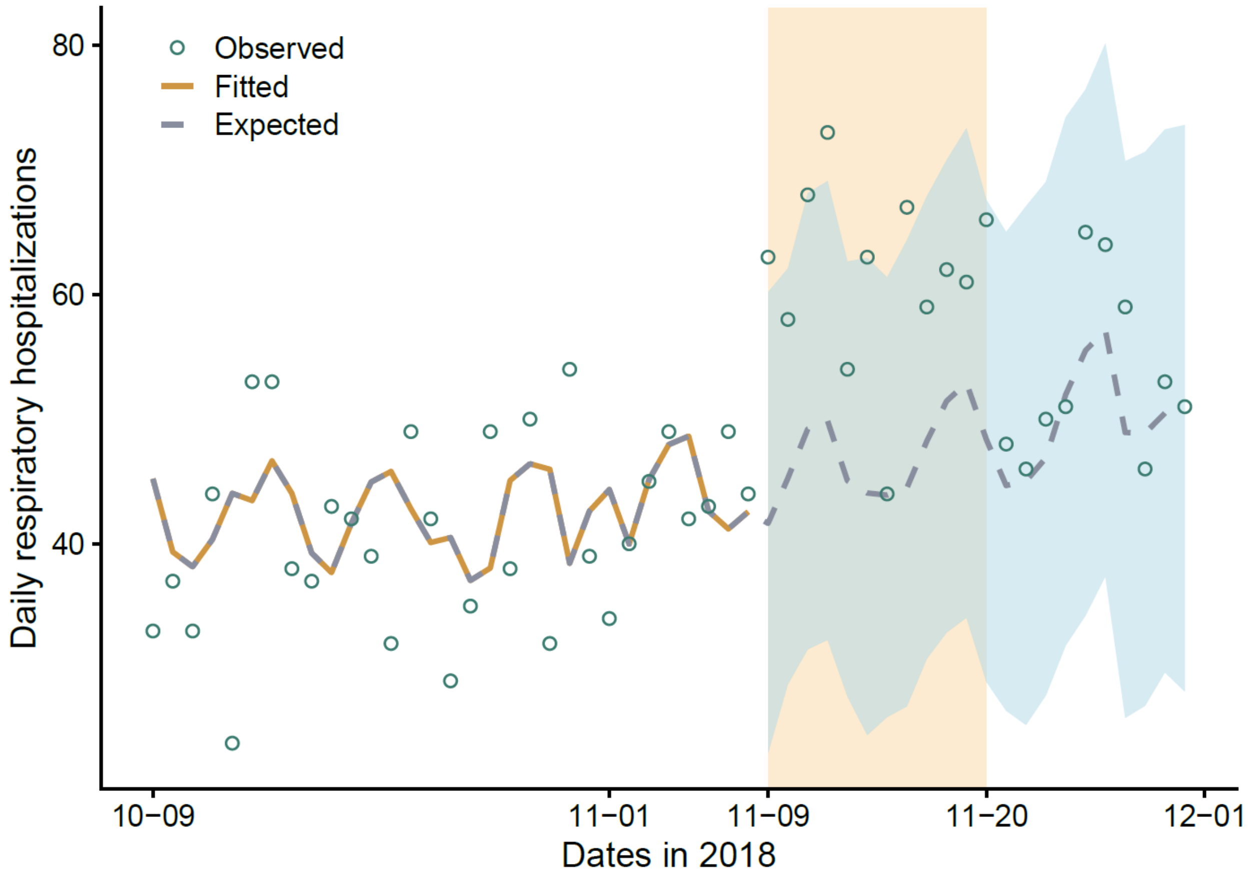

Supplementary Fig. 2. Daily observed, fitted, and expected respiratory hospitalizations from the ARIMAX model with bootstrapped prediction intervals

This figure displays the daily observed counts of respiratory hospitalizations (green circles), the fitted values from the ARIMAX model (yellow curve), and the expected values under the counterfactual scenario in which the event did not occur (dashed grey curve). The event period is highlighted by the orange rectangle. The fitted and expected curves overlap in the pre-event periods. The 95% prediction intervals were estimated by bootstrapping (1,000 times). We estimated approximately 174 excess hospitalizations during the smoke event, with wide uncertainty (95% PI: −52 to 390).

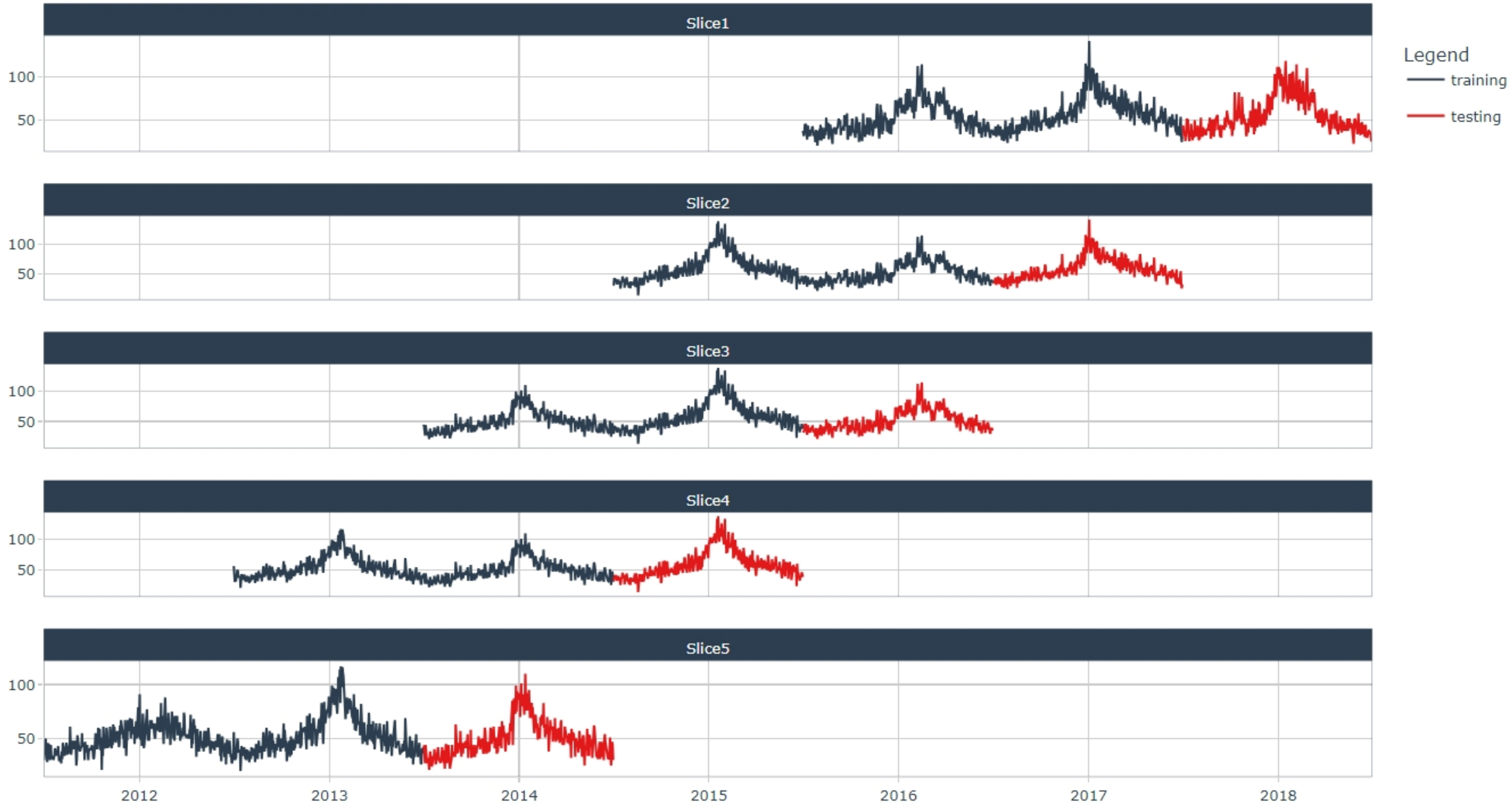

Supplementary Fig. 3. Cross-validation plan for the machine learning model.

References

1. State of California. *California Department of Health Care Access and Information*. 2024; Available from: https://hcai.ca.gov/.
2. Abatzoglou, J.T., *Development of gridded surface meteorological data for ecological applications and modelling.* Int J Climatol, 2013. **33**(1): p. 121-131.
3. Aguilera, R., et al., *A novel ensemble-based statistical approach to estimate daily wildfire-specific PM2.5 in California (2006-2020)*. Environ Int, 2023. **171**: p. 107719.
